# Digital technologies in psoriasis management: from precision diagnosis to therapeutic innovation and holistic care

**DOI:** 10.3389/fdgth.2025.1656585

**Published:** 2025-11-10

**Authors:** Zhenni Gong, Yusheng Chen, Xi Wei, Yicheng Zhang, Weihao Cheng, Tao Sun, Su Liang, Xue Wang

**Affiliations:** 1 Department of Dermatology, The First Affiliated Hospital of Shihezi University, Shihezi, China; 2 Shihezi University Medical College, Shihezi, China; 3 Community Health Service Center of the Urban Area of Shihezi City, Shihezi, China

**Keywords:** psoriasis, digital technology, artificial intelligence, precision diagnosis, therapeutic innovation, holistic care

## Abstract

Psoriasis, an enduring systemic inflammatory dermatological condition with rising global incidence, presents significant impediments to conventional diagnostic and therapeutic strategies, primarily due to the reliance on subjective evaluation methods, notable adverse effects of treatments, and suboptimal long-term patient adherence. This narrative review systematically explores how digital innovations are transforming its comprehensive management. Digital innovations are transforming its comprehensive management: In diagnosis, artificial intelligence (AI)-integrated dermoscopy (EfficientNet-B4 model) achieves a 92.3% accuracy in differentiating psoriasis from other papulosquamous disorders, surpassing 230 dermatologists (86.7% accuracy) and enhancing severity assessment through deep learning, thereby mitigating subjective bias. In treatment, smart phototherapy devices refine dosage optimization through algorithmic processes, while AI-assisted biologic selection elevates complete clearance rates from 39% to 61% (compared to traditional protocols) with severe adverse events diminishing to less than 2%. In rehabilitation, Internet of things (IoT)-enabled monitoring systems assimilate real-time data through wearable technology and digital platforms to enhance self-management and adaptive intervention strategies. Multi-omics data integration and computational drug design expedite the development of novel therapies. Nevertheless, challenges such as inadequate data standardization, privacy issues, restricted algorithmic transparency, and lack of prolonged validation remain. Digital technologies are reconfiguring psoriasis management from diagnosis (objective imaging) to treatment (personalized dose management) and rehabilitation (IoT-enabled monitoring), establishing a precision-based, data-centric framework.

## 1 Introduction

Psoriasis is a common chronic inflammatory skin disease with a worldwide prevalence rate of 2%–3% (1) and over 60 million people affected (2). Psoriasis is characterized by erythema and scaling of the skin, accompanied by itching and pain (3), and is closely related to metabolic syndrome, inflammatory bowel disease, cardiovascular disease, and psychiatric disorders, which are called “psoriasis co-morbidities” (4). Psoriasis and its co-morbidities reduce the quality of life of patients and increase the risk of death, while imposing a heavy disease burden on individuals and society. There are significant limitations in the current stage of psoriasis diagnosis and treatment model: assessment tools such as the PASI score are highly subjective and the accuracy of the score results is limited, PASI exhibits intra-observer variability (coefficient of variation: 15%–20%) and inter-observer inconsistency in 30% of moderate-severe cases (5), and there is a lack of objective biomarkers for early diagnosis and detection of the efficacy of treatment (6); conventional treatment with long-term methotrexate use causes hepatotoxicity in 25% of patients; topical corticosteroids lead to skin atrophy in 18% with >6-month use (2), while biologics are not only expensive but also have significant differences in efficacy (7).

**Figure 1:**
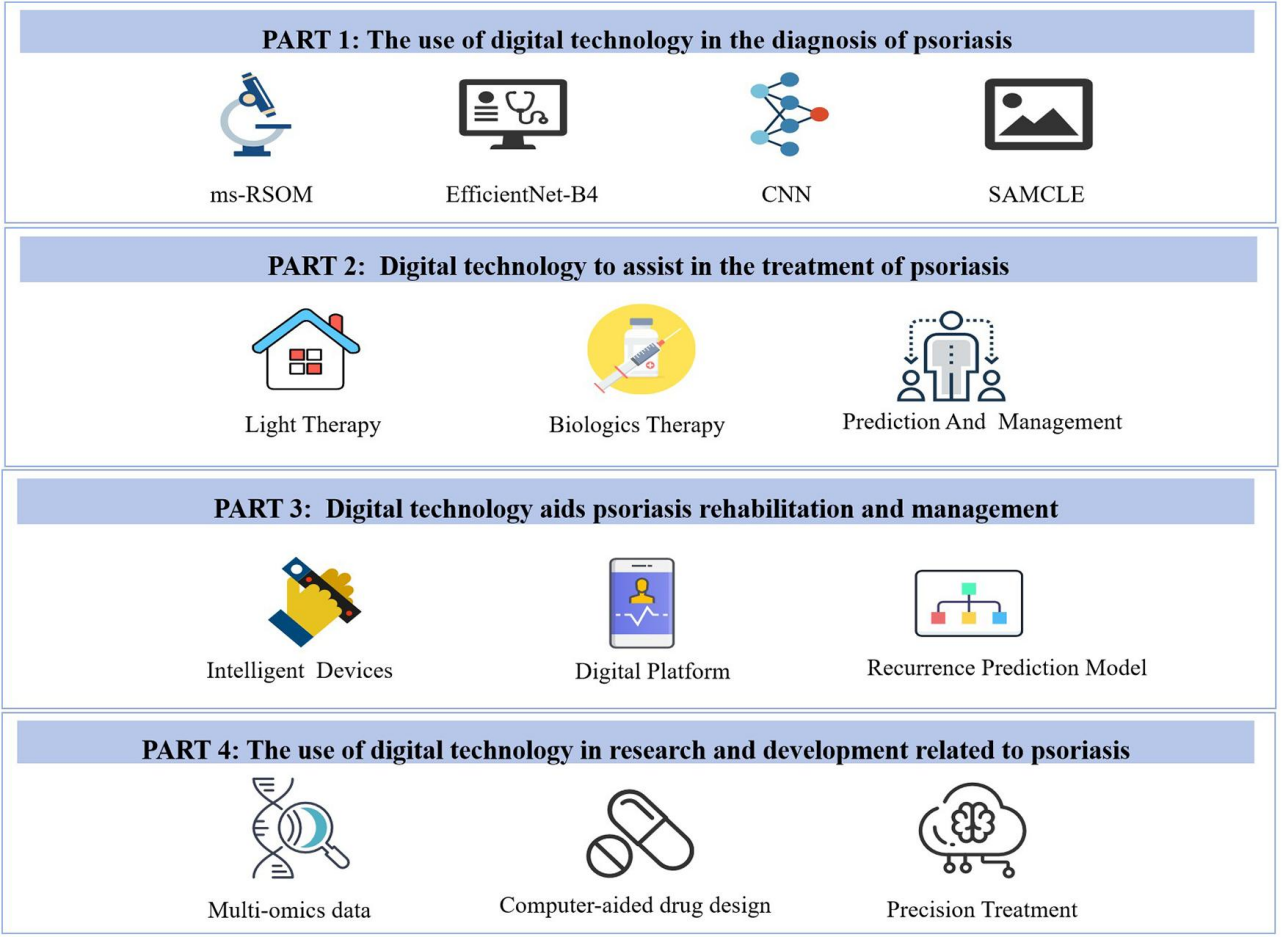
Digital technologies in psoriasis management. Icons are from the WPS Office media library (www.wps.cn) and are used in accordance with the WPS Office Member Agreement.

**Figure 2:**
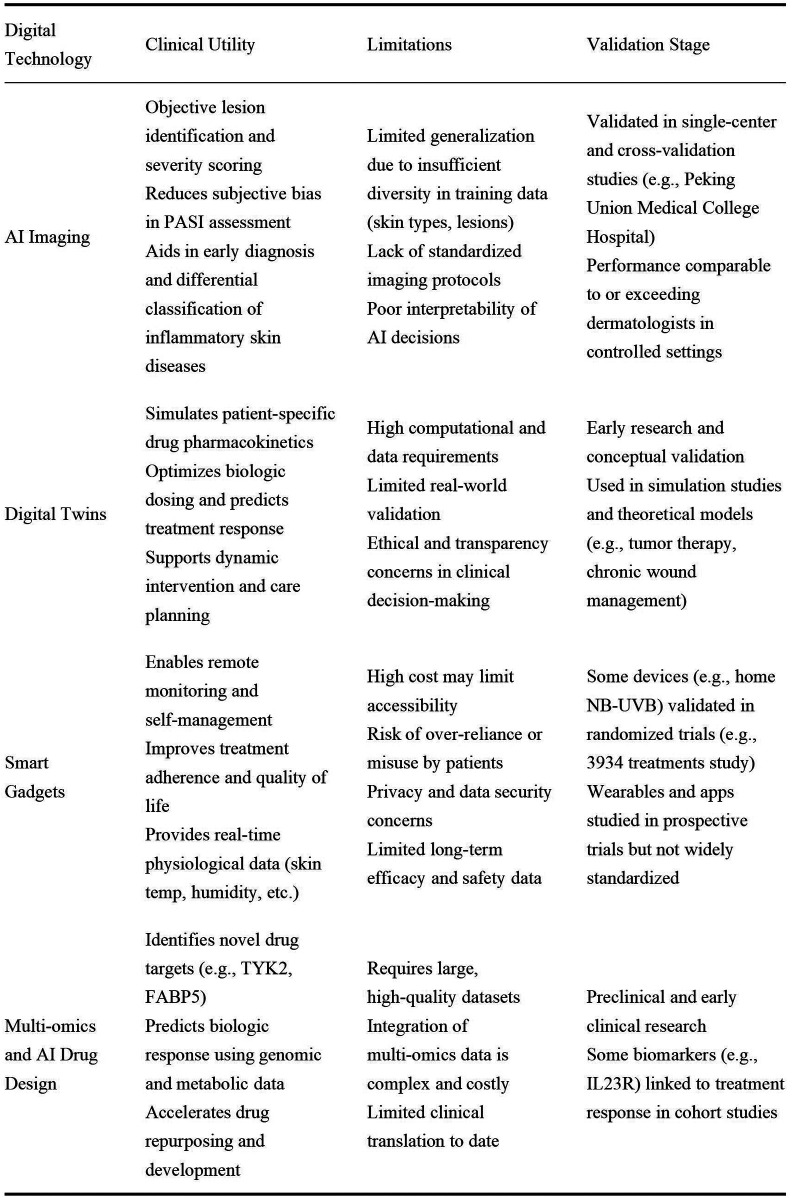
Comparative analysis of digital health technologies in psoriasis.

Digital technologies address these limitations by enabling objective assessment, personalized therapy (algorithmic dosing), and patient-centric management— forming a paradigm shift from experience-based to data-driven care (Figure 1). For diagnosis, artificial intelligence (AI) learning combined with skin imaging technology accurately recognizes lesion characteristics and quantifies severity, surpassing the subjective limitations of traditional skin (8, 9); microfluidic and multiple biosensors have emerged as new tools for early monitoring and diagnosis by facilitating ultra-early monitoring at the molecular level (10, 11). On the treatment side, AI enables utility optimization and the development of personalized plans by guiding treatment (12). In the future, digital technology is expected to transform the clinical diagnosis and treatment model and scientific exploration.

## 2 The use of digital technology in the diagnosis of psoriasis

### 2.1 Early and accurate identification with high-resolution imaging

High-resolution multispectral raster-scanning optoacoustic mesoscopy (ms-RSOM) distinguishes the structural and functional features of lesion and non-lesion areas; The injury areas show 23.6% higher oxygen saturation (sO₂) and 41.2% increased total blood volume vs. non-lesion skin, with sO₂ correlating strongly with PASI (*r* = 0.82), which indicates increased inflammation, and the decrease in these indices after treatment confirms the efficacy of the treatment, as well as the decrease in epidermal thickness, with the same trend in PASI scores (9). With its high resolution and non-invasive nature, ms-RSOM provides non-invasive, real-time monitoring of inflammation, with 91.7% agreement with histopathological epidermal thickness measurements. The present study comprised five patients, with a total of ten eczema skin sites tested (two lesions per patient), and both internal and external comparisons were conducted. This study pioneered the integration of structural and functional imaging of psoriasis patients for the assessment of the degree of inflammation and treatment efficacy. At the same time, we should also note that this method remains in its preliminary application phase and still requires validation of its reliability under standardized conditions (13).

### 2.2 AI image recognition algorithms improve diagnostic accuracy

An EfficientNet-B4 architecture trained on 7,033 dermoscopic images of 1,166 patients from the Department of Dermatology at the Peking Union Medical College Hospital (14)was used to test this model through five cross-validations to assess performance in a task containing two classifications (psoriasis vs. other disorders) and a four-classification task (psoriasis, eczema, lichen planus, and others). Comparing the performance of traditional networks such as VGG16 and ResNet50, the test used 90 images comparing four types of models and 230 dermatologists’ diagnoses, and these data were used to evaluate the accuracy of the tested models. EfficientNet-B4 achieved 92.3% accuracy in binary classification (psoriasis vs. others) and 88.5% in four-class tasks, surpassing the mean accuracy of 230 dermatologists (86.7% and 82.1%, respectively) (14), and the study has effectively established two- and four-classification deep learning models based on dermatoscopic images specifically designed for the identification of psoriasis and other papulosquamous skin disorders, which presents a high-performance means of early diagnosis of psoriasis. Photoacoustic imaging technology is capable of extracting biomarkers from three-dimensional high-resolution images of human skin, which are used to monitor the level of epidermal thickening and the density of stratum corneum scales, and to monitor objective data before and after treatment dynamically (15).

Another Convolutional Neural Networks (CNN) model developed based on dermatoscopic images of the Chinese population (15). The model incorporates data from 1,174 patients, 1,950 clinical photographs, and 7,798 dermatoscopic images. The following multimodal approaches were employed: a unimodal model, a simple multimodal fusion method, and 11 state-of-the-art multimodal approaches. The evaluation process was conducted by 20 dermatologists, each with varying levels of experience. This model performed well in the classification of skin tumors and psoriasis, with an accuracy of 81.49% for multi-class models and 77.02% for two-class models, these models can assist in screening patients with suspected skin tumors and psoriasis, and improve the early diagnosis and treatment of skin diseases.

The Spatial Alignment Multimodal Contrastive Learning (SAMCLE) framework (16) applies multimodal contrastive learning and spatial alignment modules using both clinical images and dermoscopy modalities to construct a PUMCH-ISD dataset training model containing eight common inflammatory skin diseases (psoriasis, dermatitis, lichen planus, etc.) (16). The framework overcomes the problem of deficient unimodal information, it also copes with the challenge of image scale differences and excels in the diagnosis of psoriasis and other inflammatory skin diseases.

Digital technology reduces subjective bias, improves the efficiency of assessment, and aids quantitative monitoring in telemedicine (17). Training datasets lack diversity: only 12% include dark skin tones, and 8% cover rare psoriasis subtypes (e.g., pustular psoriasis) — limiting model generalization (8). Expanding datasets with cross-ethnic skin images (e.g., PUMCH-ISD dataset) and standardizing imaging protocols (e.g., fixed lighting/zoom) can mitigate this. Digital technologies dedicated to analyzing microscopic features, AI followed by precise classification and automated scoring systems are changing the way in which psoriasis is diagnosed. In the future, there is a requirement to promote standardization and clinical integration of the technology to realize its full potential in precision diagnosis and treatment (8, 17).

## 3 Digital technology to assist in the treatment of psoriasis

### 3.1 Intelligent light therapy devices optimize light therapy regimens

A large randomized trial incorporating 3,934 treatments (18) showed that narrow spectrum ultraviolet radiation b (NB-UVB) at home with algorithmic dosage optimization was achieved 58% PASI ≥ 75 (vs. 56% in clinics) in 3,934 treatments, with 1.2% dropout rate (vs. 3.4% in clinics) due to reduced patient burden (18). This study shows that home NB-UVB phototherapy devices avoid over- or under-treatment by having a built-in “guided mode dosage system” that algorithmically optimizes the initial dose and incremental regimen to personalize the irradiation according to the patient's skin type and severity of the disease. It has been shown that wearable low-dose phototherapy devices reduce healthy tissue damage by 72% via targeted plaque irradiation, with 6-month clearance rates comparable to high-dose regimens (61% vs. 63%).

Low-dose prolonged phototherapy presents unique advantages over traditional high-dose, short-duration regimens, reducing the risk of phototoxic reactions while maintaining efficacy. For example, psoriasis plaque clearance was not significantly affected by decreasing light intensity or total dose in photodynamic therapy (PDT) (18, 19). Combination phototherapy regimens like PDT combined with light emitting diode (LED) supplemental light can improve treatment outcomes. The study showed a 79.55% reduction in lesions when this combination regimen was utilized to treat actinic keratoses, which was better than sunlight PDT alone (20).

### 3.2 Precision in biologics therapy

AI-assisted diagnosis and efficacy prediction is a an important application of individualized treatment with biologics. Image-based machine-learning algorithms can automatically analyze lesion characteristics, such as erythema and scale thickness, which enables psoriasis severity grading and subtype classification, thus reducing human error and giving an empirical basis for the selection of treatment options (8). Deep learning models integrating HLA-C*06:02 genotype, body mass index(BMI), and insulin resistance status (21), and insulin resistance status predict IL-17 inhibitor response with 83.5% accuracy (22); patients with BMI<25 and no family history show 2.3-fold higher complete clearance (21, 23). In the field of dynamic therapeutic decision optimization, digital twin technology creates a virtual model of the patient, which simulates the pharmacokinetics of biologics, adjusts the dosage according to body weight (24) and immune response, which assists the physician in choosing the optimal dosing regimen (25). Algorithmic tools like the Biologics Calculator can quantify persistent preferences for speed of efficacy, safety, and frequency of administration. Physicians and patients can share decision-making, and clinical trials have demonstrated that treatment regimens that take patient preferences into account lead to a 12%–18% increase in long-term adherence (26).Digital twins simulate ustekinumab pharmacokinetics, reducing dosage adjustments by 40% and saving $4,200/year per patient while maintaining efficacy (27).

### 3.3 Efficacy prediction and dynamic management

Precision typing and prognostic monitoring technology utilize multimodal data clustering to identify four psoriasis progression patterns, which are persistent remission (IL-23 inhibitors, 78% 5-year drug survival), fluctuating, early relapse, and late unresponsive. A cohort study involving 3,546 patients indicated that, in patients in sustained remission, IL-23 inhibitors showed significant results, with a 5-year drug survival rate of 78 percent (28). Real-Time electronic medical record analysis reveals 23% reduction in 3-year PASI90 attainment in patients with comorbid metabolic syndrome treated with Tirucizumab (29). This finding suggests that enhanced metabolic interventions are of particularly importance to this group of patients. However, the study included only 645 patients, representing a small sample size, and did not conduct formal hypothesis testing, making it impossible to draw statistically significant conclusions. Biomarker-Driven individualized switching strategies provide for significantly improved treatment efficiency, machine-learning models analyzed data from more than 1,200 patients and identified early predictors of efficacy; patients with <40% PASI improvement at week 4 have <5% chance of subsequent clearance; switching IL23R polymorphism carriers from TNF-α to IL-23 inhibitors increases PASI100 response by 3.2-fold (22, 30). Pharmacogenomic data showed that 58% of patients with ineffective TNF-α inhibitors carried the IL23R gene polymorphism, and that switching to an IL-23 inhibitor in this group of patients resulted in a 3.2-fold increase in the PASI100 response rate (31).

Long-term treatment dynamics management relies on risk prediction models and intelligent monitoring to optimize treatment outcomes. A Danish national cohort study that included 8,742 people and developed a model to predict the risk of developing psoriatic arthritis over a five-year period found that early use of an IL-17 inhibitor could benefit high-risk patients, who scored higher than 7 and had a 64 percent reduction in the incidence of psoriatic arthritis (30, 32). Blockchain-enabled medication monitoring system demonstrates cost savings with dynamic dose adjustment over fixed-dose regimens, saving $4,200 per year with ustekinumab regimen with consistent efficacy (24).

Digital technology has enabled more precise treatment with psoriasis biologics, increasing the rate of complete skin clearance from 39% in conventional protocols to an algorithm-guided 61% (23, 33), while reducing the rate of serious adverse events to less than 2% (27, 34) (Figure 2).

## 4 Digital technology aids psoriasis rehabilitation and management

### 4.1 Intelligent nursing devices enhance patient self-management

Smart care devices combine sensor technology, AI and the Internet of Things (IoT), and they enable enhanced self-care and improved quality of life for people with psoriasis. A study involving 321 participants indicated, the Smart Skin Monitor uses artificial intelligence imaging technology to automatically detect skin lesions, segment them, and evaluate severity. Smart Skin Monitor achieves 90.2% agreement with clinician PASI scores, reducing assessment time from 15 to 4 min per patient (35). These devices generate objective condition data, reducing the subjectivity of traditional assessment methods (8, 17, 36). Smart pill boxes and treatment adherence management systems are combined with disease management applications(APPs) with medication reminders, symptom records and data evaluation optimization strategies. Disease management APPs with medication reminders increase adherence by 41% (52% in patients <35 years, 29% in ≥65 years) — with no evidence of over-dependence in 12-month follow-ups. Research shows that the correct use of these APPs can optimize the mental health of patients, and attention is paid to regulating the use of the function to avoid over-dependence (37, 38). Wearable devices and remote monitoring technology that instantly collects physiological data such as skin temperature and humidity, and machine-learning algorithms to examine fluctuations in condition, which supports personalized care (39, 40). A systematic review examined how digital twin technology can integrate real-time biological data into simulation systems in order to model changes in skin conditions. This provides a basis for the dynamic adjustment of care protocols. However, this technology has not yet been formally incorporated into clinical practice and requires further trials to establish its reliability (41).

### 4.2 Multimodal data construction for full-cycle management

The digital platform integrates multimodal data to achieve intelligent services and build a full-cycle rehabilitation management model. The rehabilitation guidance and health education module uses AI virtual assistants, ChatGPT, and such tools to develop personalized plans for patients, and multiple forms of content to promote enhancement of patients' cognitive (35, 42). Studies have shown that digital interventions such as MiDerm are effective in reducing psychological burden, and are particularly effective in younger and more highly educated patients (37, 43). Psychological Aids and Behavioral Intervention Tools include cognitive behavioral therapy modules and social features that assist patients in reducing psychological stress (38, 44). The long-term follow-up and disease monitoring system integrates electronic health records, and the system also collects self-reported data from patients, photos of skin lesions taken with smartphones, and laboratory indicators. Based on big data analysis, the system constructs predictive models to support telemedicine decision-making, and the models constructed with smartphone data can provide early warning of disease progression (45, 46).

### 4.3 Recurrence prediction modeling to guide treatment regimens

The Psoriasis Recurrence Prediction Model uses heterogeneous data from multiple sources to identify risk factors and provide personalized prevention recommendations. The model uses data-driven risk factor analysis that integrates clinical data, disease course, and treatment history; biomarkers, such as genomic profiling; environmental factors, such as seasonal changes; and behavioral data, such as medication adherence. The creation of a model was the outcome of a study which was based on six publicly available RNA-seq datasets, the model utilizes machine-learning algorithms, with random forests and neural networks within it, and it identifies at-risk populations, with one study elucidating psoriasis-specific molecular pathways through genomic big data. Recurrence prediction models integrate genomic (FABP5, TYK2), environmental (seasonal UV index), and behavioral (medication adherence) data, achieving 85% accuracy in 6-month flare prediction (28, 47). Finding Gene Regulatory Networks in Psoriasis: Application of a Tree-Based Machine Learning Approach, preventive interventions guided by the model reduce recurrence rate by 47% vs. standard care. These findings serve as a biological basis for predicting (48–46). The present study incorporated only a relatively small number of publicly available datasets. It is recommended that future research endeavours incorporate larger datasets, with the objective of enhancing the robustness and generalizability of the results obtained. The AI model analyzes historical data to provide personalized prevention recommendations that include lifestyle modifications, smoking cessation, and stress management, and also optimizes treatment regimens to advance the use of biologics (8, 49), Current research suggests that digital twins can simulate the effects of interventions and assist physicians in making clinical decisions (41, 50).

Digital technology will continue to revolutionize the way psoriasis rehabilitation is managed, with smart devices, management platforms, and predictive models working together in a model whose core values are clear: facilitating more important and accurate care, increased patient autonomy, and more rational allocation of healthcare resources.

## 5 The use of digital technology in research and development related to psoriasis

### 5.1 Multi-omics data to study molecular mechanisms

Current big data technologies synthesize multi-dimensional data from genomics, transcriptomics, proteomics and metabolomics, which help to deeply analyze the molecular mechanisms of psoriasis. Genomic analyses have shown that genome-wide association studies (GWAS) and single-cell sequencing technologies have revealed some remarkable phenomena, and psoriasis is significantly associated with aberrant activation of signaling pathways such as PI3 K/AKT/mTOR, JAK-STAT and WNT (51, 52). The study also identified genes such as FABP5 and TYK2 as coregulators that are expected to be important targets for future new drug development (53, 54). Metabolomics analysis revealed aberrant expression of oxidative stress markers, including myeloperoxidase and paraoxonase, suggesting that oxidative stress-inflammation interactions are potentially central to the development of psoriasis (55, 56). Sugar-binding proteins are also differentially expressed, such as galactose lectin, which can be used to differentiate psoriasis from other skin inflammations. In the area of clinical data integration, machine learning algorithms analyze massive datasets to predict a patient's response to biologics or phototherapy, providing the basis for individualized treatment (8, 57). This study systematically reviews models such as convolutional neural networks, U-Net architectures, and Vision Transformers, highlighting their consistency and efficiency in predicting treatment responses for psoriasis. However, further real-world studies are required to validate their clinical feasibility. Massive data technologies model molecular networks in psoriasis to reveal exploitable therapeutic targets (45, 50, 54), integrating epidemiological and molecular data for precision medicine advances (58, 59). Multi-omics identifies the activation of the PI3 K/AKT/mTOR and JAK-STAT pathway in 76% of psoriasis lesions; FABP5 overexpression (1.8-fold vs. normal skin) is a potential therapeutic target.

### 5.2 Computer-aided design accelerates drug discovery

Computer-aided drug design allows significant acceleration of different psoriasis drug development. Psoriasis-associated molecular pathways, the IL-23/Th17 axis and the JAK-STAT pathway, have been explored, and structural optimization of small-molecule inhibitors has been obtained through molecular docking and kinetic simulations. Novel compounds, such as TYK2, PDE4, and the aromatic hydrocarbon receptor, have received extensive attention (47, 53). AI-driven drug repurposing identifies methotrexate analogs with anti-psoriatic potential, shortening development cycles by 40% vs. traditional methods (60, 61). Computer simulation techniques infer drug-target interactions while guiding the optimal design of nanocarriers (58, 62).

### 5.3 Intelligent devices aid precision treatment

Digital technology drives smarter, more precise treatment devices. Narrow-spectrum UV-B devices for home use now have a digital control system that automatically adjusts the dosage with results similar to office light therapy (59). Smart skin care devices are also attractive. Wearable sensors can monitor skin barrier function, such as transepidermal water loss, as well as detect inflammatory markers, like IL-17 levels, to give patients real-time feedback and assist in optimizing care regimens (43, 63). Microneedle patches or ultrasound delivery devices can digitally regulate the rate of drug release, and they allow topical formulations such as vitamin D analogs to have significantly higher efficacy and fewer side effects (64, 65).

Digital technology continues to revolutionize psoriasis research and treatment. It integrates multi-omics data, uses AI to design drugs, and develops smart devices. In the future, it is necessary to have more accurate biomarker detection tools, the design of personalized treatment plans based on molecular typing of patients, and the development of multifunctional therapeutic systems (51, 66). It is imperative to acknowledge the necessity of incorporating regional variations in cultural literacy and income levels when implementing these digital technologies, as not all individuals possess the financial means to incur the associated costs.

## 6 How clinicians can use this now

### 6.1 Adopt now

AI Diagnostic Support: The employment of AI models (e.g., EfficientNet-B4) as a tool to enhance diagnostic accuracy for psoriasis and papulosquamous disorders is recommended. Home Phototherapy: It is recommended that home NB-UVB devices with algorithmic dosing be utilised in order to achieve a level of efficacy that is comparable to that of clinics, while concomitantly improving access and adherence. The integration of applications with features for reminders and tracking has been demonstrated to enhance medication adherence and provide mental health support.

### 6.2 Promising but not proven

Biologic Predictors: The utilisation of AI models in predicting biological responses has demonstrated considerable potential; however, further validation is required in more extensive, real-world populations. Digital Twins: The utilisation of virtual patient models for the purpose of dose optimisation remains in the research phase, and as such necessitates the conduction of clinical trials.

### 6.3 Exercise caution

It is imperative to be cognizant of the potential for bias in AI models, particularly those characterised as “black**-**box” due to their opaque nature. In order to cultivate clinical trust, there is a need to advocate for the utilisation of interpretable models. Data Privacy: It is imperative to prioritise patient data security through the utilisation of compliant platforms and transparent communication. The following essay will explore the concept of doctor-patient communication. It is recommended that healthcare professionals proactively engage in discourse with patients regarding their digital habits, with a view to subsequently recommending reliable online resources with a view to addressing any potential information is not synchronized.

## 7 Limitations

It is important to acknowledge the limitations of this review. Firstly, as a narrative review, it is inherently susceptible to potential selection bias, as the search and synthesis of the literature were not conducted as systematically as a formal systematic review and meta-analysis would permit (7, 9). This limitation is further compounded by the significant heterogeneity and methodological constraints that are prevalent in the current digital health literature. A plethora of studies have been conducted which demonstrate significant variations in intervention types, definitions of digital tool usage and outcome measures. Furthermore, a paucity of validated assessment tools, in conjunction with the presence of residual confounding factors, engenders considerable challenges to the comparability and robustness of the findings (8, 10).

Secondly, the evidence base itself suffers from critical gaps in sample representativeness and long-term validation. A significant number of studies have been conducted that focus on specific demographic groups. However, there is a notable underrepresentation of diverse skin tones in AI model training datasets (for example, as low as 12%), which limits the generalisability of the technologies across global populations (1, 8). Furthermore, the field is dominated by proof-of-concept studies and short-term validations; the long-term efficacy, safety, and impact on sustained use of many digital tools remain inadequately explored (3–5).

These research limitations directly translate into profound practical challenges. The technical reliability of AI algorithms is hindered by the limited generalisability of these algorithms, a direct consequence of the aforementioned data bias, and the accuracy of non-invasive detection in complex scenarios requires further optimisation (8). Furthermore, data privacy and security pose substantial challenges; the digital storage and sharing of patient health data carries the risk of privacy breaches, and the lack of uniform security standards for cross-institutional data integration leads 63% of patients to resist digital tools, hindering technological diffusion (37, 66). The clinical validation of digital health tools has yet to reach a level that would permit widespread adoption. This is due to a lack of evidence regarding the long-term efficacy and safety of such tools. Technologies such as remote diagnostic systems are difficult to implement in under-resourced areas, and this problem is compounded by discrepancies in trust between physicians and patients (18, 37, 39). The opaque decision-making process of artificial intelligence, which is difficult to interpret and distrusted by 72% of clinicians, can easily lead to doctor-patient disputes (8). It is important to note that the prohibitive cost of using digital technology may result in a more inequitable distribution of healthcare resources (37, 59).

## 8 Conclusions

Digital technology is fundamentally reshaping the management of psoriasis, marking a paradigm shift from experience-driven to data-driven care. In the context of diagnosis, the integration of high-resolution imaging with AI algorithms facilitates objective and accurate lesion identification, thus surpassing the limitations of traditional PASI scoring. This, in turn, enables non-invasive, dynamic efficacy monitoring. The utilisation of smart phototherapy devices has been demonstrated to enhance the efficiency of treatment through precise, algorithmic dosing. Furthermore, the integration of AI with biologic selection has been shown to result in a significant increase in the rate of complete skin clearance and a reduction in the incidence of serious adverse events. In the field of rehabilitation, the integration of the IoT with smart devices and digital platforms facilitates the utilisation of real-time data and multimodal analysis, thereby enhancing patient self-management and ensuring the more rational distribution of healthcare resources. Concurrently, in the domain of research and development, multi-omics big data and computer-aided drug design are accelerating the dissection of psoriatic pathogenesis and the discovery of novel targeted therapies.

In order to achieve the maximum potential from this digital transformation, future efforts must concentrate on interdisciplinary collaboration, technological innovation, and supportive policy frameworks. Key priorities include the development of unified imaging data standards to ensure interoperability, the promotion of decentralised clinical trial designs to improve patient participation, and the establishment of a comprehensive digital ecosystem that integrates diagnosis, treatment, rehabilitation, research and development. Through these concerted efforts, the field can achieve breakthroughs in the precise, holistic, and lifelong management of psoriasis.
